# Drug–drug interactions between epidermal growth factor receptor tyrosine kinase inhibitors and rivaroxaban in vitro and in vivo

**DOI:** 10.1371/journal.pone.0322303

**Published:** 2025-06-03

**Authors:** Dongxu Wang, Shuanghu Wang, Hualan Wu, Peiwu Geng, Yang An, Xiaoyue Zhou, Minghui Du, Yuwei Li, Jia Chong, Yingying Li, Fang Wang, Zebei Lu, Yu Wang, Jiefu Yang, Chuanbao Li, Dapeng Dai, Hao Chen

**Affiliations:** 1 Department of Cardiovascular, Beijing Hospital, National Centre of Gerontology, Beijing, China; 2 Arrhythmia Center, Fuwai Hospital, Chinese Academy of Medical Sciences, National Center for Cardiovascular Diseases, Beijing, China; 3 Laboratory of Clinical Pharmacy, The Sixth Affiliated Hospital of Wenzhou Medical University, The People’s Hospital of Lishui, Lishui, Zhejing, China; 4 Department of Clinical Laboratory, Beijing Hospital, National Center of Gerontology, Beijing, China; 5 The Key Laboratory of Geriatrics, Beijing Institute of Geriatrics, Beijing Hospital, National Centre of Gerontology, Beijing, China; Alexandria University, EGYPT

## Abstract

**Background:**

Tyrosine kinase inhibitor (TKI) and rivaroxaban co-administration is common for patients with cancer and venous thromboembolism. However, the drug–drug interactions (DDIs) between epidermal growth factor receptor (EGFR) TKIs and rivaroxaban remain uncertain.

**Methods:**

DDIs were investigated *in vitro* and *in vivo*. *In vitro* experiments were conducted using rat liver microsomes, and rivaroxaban metabolites were tested to identify the two TKIs that exhibit the most significant DDIs. The type of inhibition was investigated using Lineweaver-Burk plots. For *in vivo* experiments, eighteen rats were randomly divided into three groups and pretreated with CMC-Na together with avitinib or gefitinib, or with CMC-Na alone for 7 days. On day 8, rivaroxaban was orally administered to each group. Blood samples were collected at various time points, and plasma rivaroxaban was quantified. Molecular docking was performed to explore the mechanism of DDIs.

**Results:**

Avitinib and gefitinib showed the most potent inhibitory effects among multiple EGFR TKIs and inhibited rivaroxaban metabolism in a mixed model of noncompetitive and uncompetitive inhibition. The area under the drug-time curve and maximum plasma concentration of rivaroxaban were significantly higher following avitinib and gefitinib pretreatment, while the apparent volume of distribution and clearance rates were significantly lower. Our molecular docking analysis revealed that these two drugs may inhibit rivaroxaban metabolism by overlapping with its binding site on CYP3A4 and CYP2D6.

**Conclusion:**

These findings confirm the presence of DDIs between EGFR TKIs and rivaroxaban. Avitinib and gefitinib significantly inhibit rivaroxaban metabolism, and their co-administration may aggravate the risk of bleeding.

## 1 Introduction

Venous thromboembolism (VTE) is a prevalent complication and an important cause of morbidity and mortality among patients with cancer [1,2]. Cancer is considered a significant risk factor for VTE, and approximately 20–30% of VTE incidents occur in patients with cancer [3–5]. Consequently, patients with cancer often require anticoagulant therapy to prevent and manage VTE. However, in routine clinical practice, anticoagulant treatment tends to increase the risk of bleeding. With increasing clinical evidence, direct oral anticoagulants (DOACs) have become first-line drugs for the treatment of VTE in patients with cancer and could be co-administered with antineoplastic agents [1,2]. Compared with traditional anticoagulation methods (low molecular weight heparin and warfarin), DOACs do not require subcutaneous administration or frequent laboratory monitoring. Furthermore, DOACs exhibit superior anticoagulation efficacy and a comparable or reduced risk of bleeding compared with those of traditional methods [6,7].

Rivaroxaban, an oral direct factor Xa inhibitor, is widely used as an anticoagulant to manage thrombotic events in various diseases [8,9]. International guidelines recommend rivaroxaban as an option for VTE treatment in patients with cancer [1,2,10]. Although frequent monitoring, such as that required for warfarin, is not necessary for DOACs, various enzymes involved in DOAC transport and metabolism, and the resulting drug–drug interactions (DDIs), still pose challenges in the clinical application of DOACs [11,12]. Rivaroxaban is transported *in vivo* by P-glycoprotein (P-gp) and breast cancer resistance protein (BCRP) (encoded by ABCB1 and ABCG2, respectively), which are metabolized by multiple cytochrome P450 (CYP450) enzymes, mainly CYP3A4, CYP2J2 and CYP2D6 [13–15]. Medications that affect rivaroxaban transport and metabolism may affect its pharmacokinetics and pharmacodynamics, altering its blood concentration and increasing the risk of thrombosis or bleeding [11,16,17].

Tyrosine kinase inhibitors (TKIs) inhibit tyrosine-mediated tumor cell proliferation, they have shown promising anti-tumor effects against various hematological and solid tumors [18,19]. Epidermal growth factor receptor (EGFR) TKIs have become first-line treatment options for advanced EGFR mutation-positive non-small-cell lung cancer (NSCLC) [20]. As with rivaroxaban metabolism, TKI metabolism involves numerous drug transporters and CYP450 enzymes, such as ABCB1, ABCG2, CYP3A4 and CYP2J2 [21,22]. The similarity in drug metabolic pathways raises the possibility of DDIs between TKIs and rivaroxaban, which could result in inadequate drug efficacy or increased occurrence of adverse events.

With the increasing clinical use of DOACs for VTE management in cancer patients, the potential interactions between antineoplastic agents and DOACs have become a significant concern in clinical practice. A study published in 2020, which examined over 250 antineoplastic agents and anticoagulants in a DDI database, demonstrated that there may be clinically relevant DDIs between rivaroxaban and TKIs [23]. These interactions present a challenge for anticoagulant therapy in patients with cancer. In recent years, several studies have found that a variety of TKIs have the potential to interact with rivaroxaban and inhibit the drug metabolism of rivaroxaban *in vitro* and *in vivo* [21,24,25]. DDIs may significantly affect pharmacokinetics of rivaroxaban, leading to an increased risk of anticoagulant overdose and bleeding. Lung cancer patients treated with EGFR TKIs also face an increased risk of thrombosis. Therefore, oral anticoagulants such as rivaroxaban are required for the treatment and prevention of thrombotic events. Given the potential for DDIs, it is crucial to consider both safety and efficacy when combining EGFR TKIs with rivaroxaban. However, research on DDIs between EGFR TKIs and DOACs is lacking. The aim of this study was to examine the potential DDIs between rivaroxaban and EGFR TKIs, assess the impact of EGFR TKIs on the metabolism of rivaroxaban *in vivo* and *in vitro*, predict the safety of rivaroxaban anticoagulation in cancer patients undergoing EGFR TKI treatment, and offer guidance and support for anticoagulant therapy in these patients.

## 2 Materials and methods

### 2.1 Chemicals and reagents

Rivaroxaban was purchased from J&K Scientific Ltd. (Beijing, China). Rivaroxaban metabolite M-2 (Lot No. 3530-047A1) was obtained from TLC Pharmaceutical Standards Ltd. (Newmarket, Ontario, Canada). Avitinib, gefitinib, dacomitinib, afatinib, lapatinib, neratinib, erlotinib, and zorifertinib were purchased from Lingyi Biology Scientific, Ltd. (Guangzhou, China). Midazolam was obtained from the Tianjin Golden York Pharmaceutical Co. (Tianjin, China). Nicotinamide adenine dinucleotide phosphate (NADPH) was purchased from Roche Pharmaceuticals, Ltd. (Basel, Switzerland). Chromatography-grade acetonitrile and formic acid were obtained from Merck GmbH (Darmstadt, Germany) and Sigma-Aldrich (St. Louis, MO, USA), respectively. All other chemicals were of analytical grade or higher. Rat liver microsomes (RLMs) were prepared in our laboratory according to the previously reported differential centrifugation method [26].

### 2.2 Animals

Eighteen male Sprague–Dawley (SD) rats weighing 300 ± 20 g were provided by the Experimental Animal Center of Wenzhou Medical University (Wenzhou, China) and kept under controlled conditions at a temperature of 23 ± 2 °C, relative humidity of 50 ± 10%, and 12 h light–dark cycle. Eighteen male SD rats were randomly assigned to three groups: avitinib, gefitinib, and control, with six rats per group. Prior to the experiment, the rats were not administered any other medications and fasted for 12 h. The experimental procedures and protocols adhered to Animal Ethics standards and were approved by the Animal Care and Use Committee of Wenzhou Medical University (approval no. wydw2019–650). All rats were anesthetized with sodium pentobarbital (40mg/kg, i.p) to relieve pain. The rats were killed through cervical dislocation.

### 2.3 EGFR TKI screening

To obtain an overall view of the potential DDIs between multiple EGFR TKIs and rivaroxaban, we used a 200 μL incubation system comprising 100 mM potassium phosphate buffer (pH 7.4), 0.5 mg/mL RLMs, an appropriate dose of rivaroxaban, 1 mM NADPH, and 100 μM EGFR TKI (avitinib, gefitinib, dacomitinib, afatinib, lapatinib, neratinib, erlotinib, or zorifertinib). The system was preincubated at 37 °C for 5 min, followed by the addition of NADPH (1 mM) to initiate the reaction. After incubating for an additional 30 min, a 200 μL of acetonitrile solution containing 200 ng/mL midazolam was introduced. The mixture was vortexed for 30 s, centrifuged at 13000 rpm for 5 min, and the supernatant was removed. Three samples were collected for each group. Rivaroxaban and its major metabolite M-2 in RLMs were detected by ultra performance liquid chromatography coupled with mass spectroscopy (UPLC–MS/MS), using a previously reported method [25]. The ratio of rivaroxaban metabolite formation in the incubation system with TKIs to that without inhibitors was used to represent inhibitory activity. The two drugs with the greatest inhibitory activity (avitinib and gefitinib) were selected for subsequent experiments *in vitro* and *in vivo*.

### 2.4 *In vitro* inhibitory effects of avitinib and gefitinib on rivaroxaban in RLMs

A 200 μL incubation system was established to determine the Michaelis–Menten constant (K_m_) for rivaroxaban, which consisted of 100 mM potassium phosphate buffer (pH 7.4), 0.5 mg/mL RLMs, and varying concentrations of rivaroxaban (0.5, 1, 2.5, 5, 10, 25, 50, or 100 μM). Three samples from each group were incubated, centrifuged, and separated as described earlier. The LC–MS/MS method was used to detect rivaroxaban metabolites. K_m_ was calculated by applying the Michaelis–Menten equation using Prism v. 9.0 (GraphPad Software, MA, USA).

Similarly, another 200 μL incubation system was used to calculate the half maximal inhibitory concentration (IC_50_) values of avitinib and gefitinib, comprising 100 mM potassium phosphate buffer (pH 7.4), 0.5 mg/mL RLMs, rivaroxaban at K_m_ concentration, and a concentration gradient of avitinib or gefitinib (0.01, 0.1, 1, 5, 10, 50, or 100 μM). Thereafter, the systems were incubated, centrifuged, and separated. Each group consisted of three parallel samples, with samples lacking an inhibitor serving as controls. The IC_50_ values were determined using Prism to estimate the inhibition rates of avitinib and gefitinib against rivaroxaban.

To gain a deeper understanding of the inhibitory mechanisms of avitinib and gefitinib on rivaroxaban, rivaroxaban was used at 0.25×, 0.5×, 1×, or 2× K_m_ concentrations in various reaction systems. In addition, avitinib and gefitinib at concentrations of 0×, 0.25×, 0.5×, 1×, or 2× IC_50_ were added to each group. The UPLC–MS/MS method was employed to quantify the rivaroxaban metabolites. K_i_ and αK_i_ values were computed, and the inhibitory mechanism was examined using Lineweaver–Burk plots.

### 2.5 Pharmacokinetic effects of avitinib and gefitinib on rivaroxaban *in vivo*

Prior to the experiment *in vivo*, the six rats in the avitinib group were administered 30 mg/kg avitinib and 0.5% sodium carboxymethyl cellulose (CMC-Na) solution by gavage daily for 7 d. Similarly, six rats in the gefitinib group were administered 25 mg/kg gefitinib and 0.5% CMC-Na solution, whereas the control group was administered only equal amounts of 0.5% CMC-Na solution during the pretreatment period. On the day of the experiment, all three groups were administered avitinib, gefitinib, or 0.5% CMC-Na solution by gavage, and rivaroxaban (10 mg/kg) was administered 30 min later. Blood (0.3 mL) was collected from the rats’ tail vein at 0.1, 0.25, 0.5, 1, 2, 3, 4, 6, 8, and 12 h after rivaroxaban administration, and centrifuged at 4000 rpm for 5 min. The supernatant was collected and stored at −80 °C. After thawing, 50 μL of plasma was extracted; 100 μL of acetonitrile solution containing midazolam (200 ng/mL) was then added to induce protein precipitation. After vortexing for 30 s and centrifuging at 13,000 rpm for 5 min, the supernatant was discarded. The plasma concentration of rivaroxaban was determined using UPLC–MS/MS. The mean plasma concentration–time profiles of rivaroxaban in the different groups were generated using Prism. The pharmacokinetic parameters of rivaroxaban included maximum plasma concentration (C_max_), time taken to reach maximum plasma concentration (T_max_), apparent volume of distribution (Vz/F), area under the drug–time curve (AUC), elimination half-life (t1/2), clearance rate (CLz/F), and mean residence time (MRT).

### 2.6 Molecular docking studies

Several human CYP450 enzymes that may affect rivaroxaban metabolism were selected for molecular docking analysis [27]. The three-dimensional crystal structures of CYP1A2, CYP2A6, CYP2B6, CYP2D2 and CYP3A4 were generated from Protein Data Bank (https://www.rcsb.org/). As the crystal structure of CYP2J2 has not been published, we developed a structural model of CYP2J2 by homologous simulation based on the three-dimensional structure of the CYP2 family, using the method reported by Xu et al. [28]. The molecular structures of avitinib, gefitinib, and rivaroxaban were obtained from PubChem (https://pubchem.ncbi.nlm.nih.gov). AutoDockTools v.1.5.7 (Center for Computational Structural Biology, CA, USA) was used for the preprocessing of target proteins and small molecules, and AutoDock Vina v.1.2.0 (Center for Computational Structural Biology) was used for molecular docking verification. To evaluate the binding between small molecules and target proteins, the binding energy (BE) was calculated using PYMOL v. 2.4 (Schrödinger, NY, USA).

### 2.7 Statistical analysis

All assays were performed in triplicate, and the results are expressed as mean ± standard deviation. DAS software v. 3.2.8 (BioGuider Co., Shanghai, China) was used to fit and calculate the pharmacokinetic parameters of rivaroxaban. SPSS v. 25.0 (IBM, NY, USA) was used for data processing. Student’s *t*-tests were used to detect differences between groups. Differences with *p* < 0.05 were considered statistically significant.

## 3 Results

### 3.1 Screening EGFR TKI with the most significant DDIs

**Fig 1:**
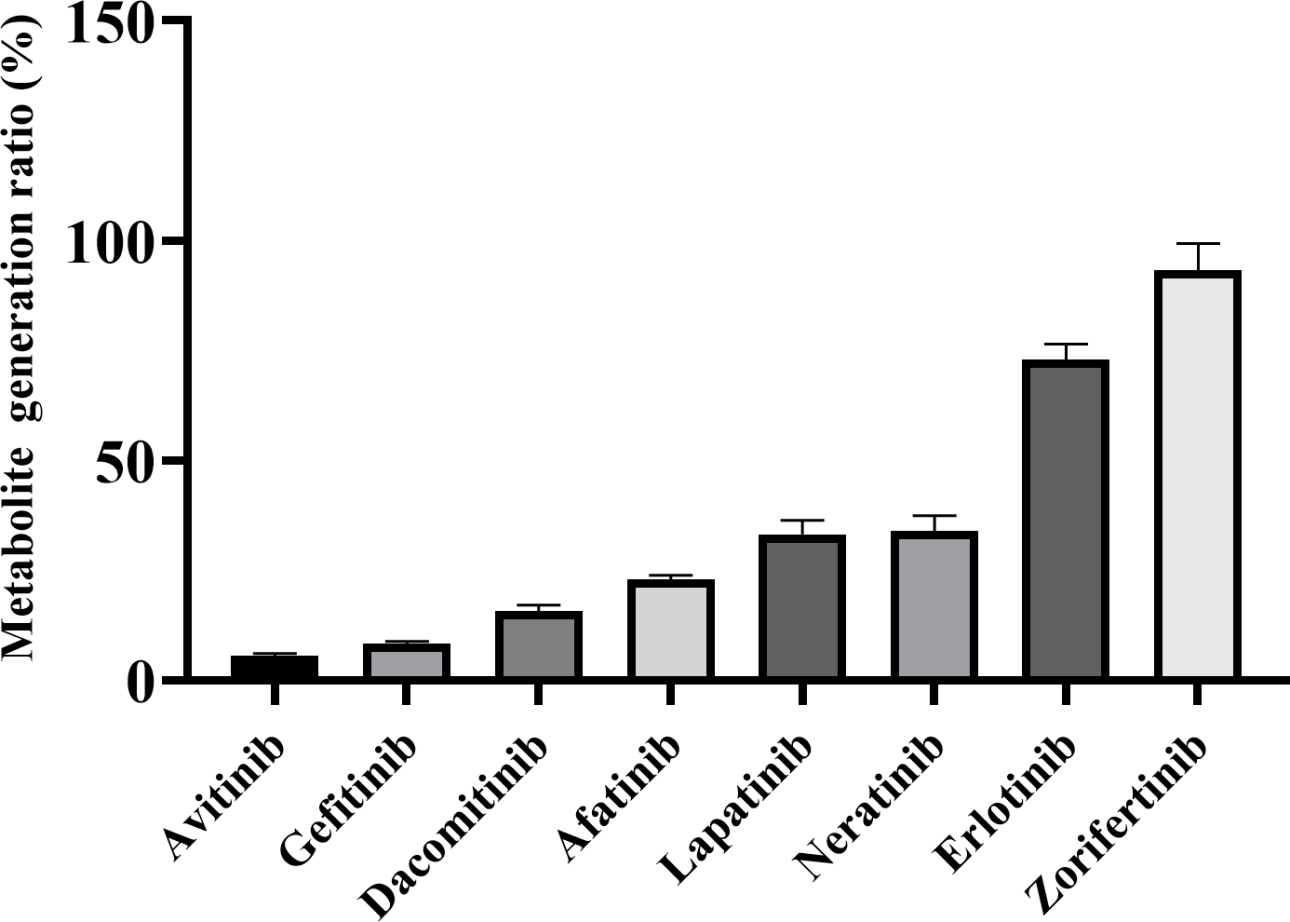
Impact of different epidermal growth factor receptor (EGFR) tyrosine kinase inhibitors (TKIs) on the metabolite generation rate of rivaroxaban.

In studies comparing the impact of different EGFR TKIs on rivaroxaban metabolism, avitinib and gefitinib were found to have potential significant DDIs with rivaroxaban. Fig 1 shows the impact of adding different EGFR TKIs to the RLMs incubation system on the generation rate of the rivaroxaban metabolite. Six types of EGFR TKIs, avitinib, gefitinib, dacomitinib, afatinib, lapatinib, and neratinib significantly inhibited rivaroxaban metabolism: the metabolite generation rate was significantly lower following TKI treatments compared to that of the control. The co-administration of these agents may elevate the risk of bleeding in patients receiving anticoagulation with rivaroxaban. Notably, avitinib and gefitinib exhibited the strongest inhibitory effects, with ratios of the metabolite concentration in these groups less than 10% (averaging 5.6% and 8.3%, respectively), relative to the control. Therefore, these two drugs were selected for subsequent DDI experiments *in vitro* and *in vivo*.

### 3.2 *In vitro* studies of avitinib and gefitinib on rivaroxaban metabolism

**Fig 2:**
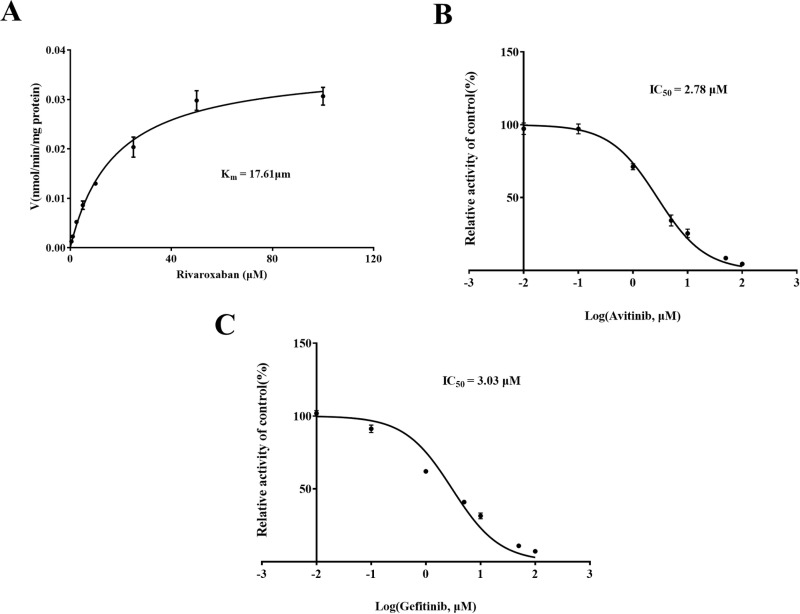
Michaelis–Menten kinetics of rivaroxaban (A) and inhibitory concentration (IC_50_) values of avitinib (B) and gefitinib (C) in rat liver microsomes (RLMs).

**Fig 3:**
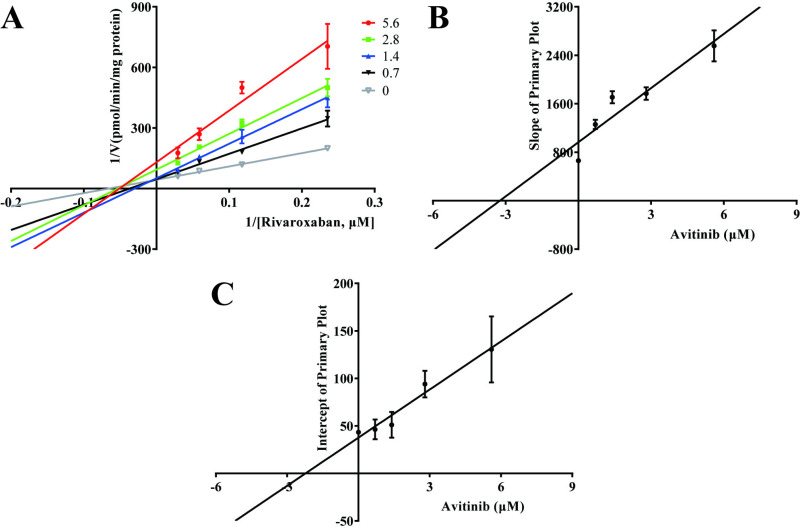
Inhibitory mechanism of avitinib on rivaroxaban. (A) Lineweaver–Burk plots for avitinib inhibition of rivaroxaban in rat liver microsomes (RLMs). (B) Slope of the primary plot. (C) Intercept of the primary plot. Data shown are the mean ± SD of triplicate experiments.

**Fig 4:**
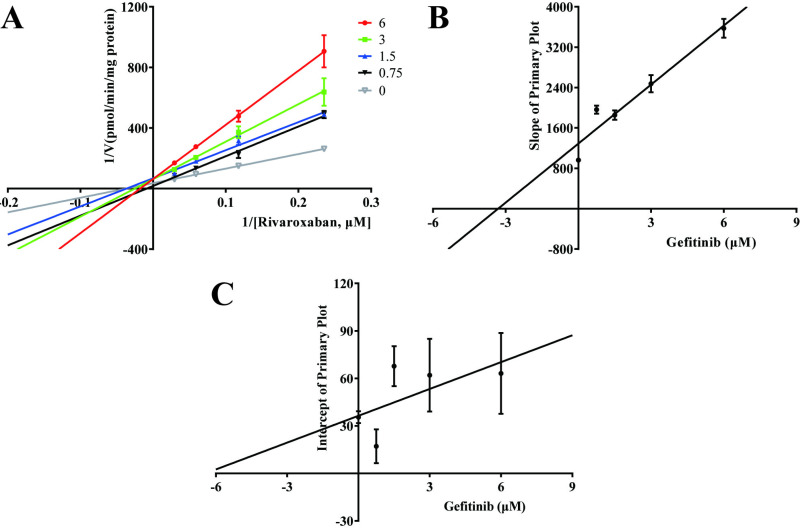
Inhibitory mechanism of gefitinib on rivaroxaban. (A) Lineweaver–Burk plots for gefitinib inhibition of rivaroxaban in rat liver microsomes (RLMs). (B) Slope of the primary plot. (C) Intercept of the primary plot. Data shown are the mean ± SD of triplicate experiments.

To further elucidate the possible mechanisms underlying the impact of avitinib and gefitinib on the metabolism of rivaroxaban, the Michaelis-Menten equation was employed to determine the enzyme kinetic parameter K_m_ of rivaroxaban (17.61μM). As shown in Fig 2, the IC_50_ values of avitinib and gefitinib were 2.78 μM and 3.03 μM, respectively, suggesting that both TKIs have a strong inhibitory effect on rivaroxaban *in vitro*, indicating a significant risk of DDIs when co-administered with rivaroxaban. A Lineweaver–Burk plot was used to investigate the mechanisms whereby avitinib and gefitinib inhibit rivaroxaban. Based on the changes in the vertical axial intercept (1/V_max_) and horizontal axial intercept (−1/K_m_) within each group, avitinib acted as a mixed uncompetitive and noncompetitive inhibitor of rivaroxaban in RLMs, with an inhibitory constant (K_i_) of 3.237 μM and a binding constant (αK_i_) of 2.225 μM (Fig 3). Similarly, gefitinib was identified as a mixed inhibitor of rivaroxaban, with K_i_ = 3.312 μM and αK_i_ = 6.436 μM (Fig 4).

### 3.3 *In vivo* effects of avitinib and gefitinib on rivaroxaban pharmacokinetics

**Fig 5:**
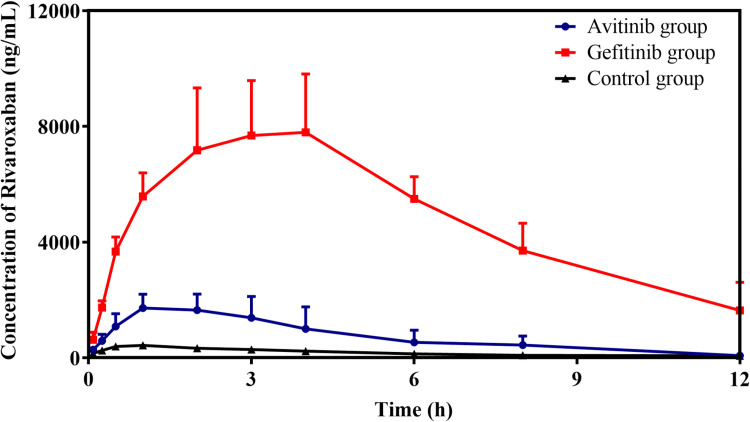
Mean plasma concentration–time profiles of rivaroxaban in the avitinib, gefitinib, and control groups (*n* = 6, mean ± SD).

Fig 5 shows the mean plasma concentration–time profiles of rivaroxaban in the different groups. The pharmacokinetic parameters are provided in Table 1. The rivaroxaban pharmacokinetic parameters were significantly higher under co-administration with avitinib and gefitinib (AUC_(0–t)_, AUC_(0–∞)_, and C_max_; *p* < 0.05) than in the control. In the avitinib and gefitinib groups, the AUC_(0–t)_ was 4.0- and 25.8-fold higher, AUC_(0–∞)_ 4.1- and 33.3-fold higher, and C_max_ 4.1- and 18.8-fold higher, respectively, than those of the control. In addition, MRT_(0–t)_ and T_max_ were significantly higher in the gefitinib group than they were in the control. However, the Vz/F and CLz/F values were significantly reduced in the avitinib and gefitinib groups. The values of Vz/F and CLz/F were reduced by 68% and 76%, respectively, in the avitinib group, and both values were reduced by 92% and 97% in the gefitinib group. These results demonstrated that pretreatment with avitinib and gefitinib inhibit the metabolism of rivaroxaban *in vivo*.

**Table 1:** Pharmacokinetic parameters of rivaroxaban in the avitinib, gefitinib, and control groups.

Pharmacokinetic parameters	Control group	Avitinib group	Gefitinib group
AUC_(0–t)_ (μg/L·h)	2247.33±853.03	8995.48±3739.25*	57981.18±5886.41*
AUC_(0–∞)_ (μg/L·h)	2280.40±869.60	9347.38±3843.72*	76028.02±20846.91*
MRT_(0–t)_ (h)	3.79±1.08	3.79±0.66	4.90±0.47*
MRT_(0–∞)_ (h)	3.95±1.05	4.26±1.12	8.23±3.61
t_1/2_ (h)	1.61±0.61	2.20±1.07	4.81±2.70
T_max_ (h)	0.83±0.26	1.33±0.82	3.17±0.98*
Vz/F (L/kg)	11.49±2.63	3.72±2.00*	0.87±0.28*
CLz/F (L/h/kg)	4.85±1.50	1.18±0.34*	0.14±0.03*
C_max_ (μg/L)	441.22±81.78	1814.82±618.90*	8310.60±2098.20*

AUC, area under the plasma concentration–time curve; CL, plasma clearance; C_max_, maximum plasma concentration; MRT, mean residence time; t_1/2_, elimination half-life; T_max_, time taken to reach maximum plasma level.

*n* = 6 in each group; data were expressed as mean ± standard deviation (SD).

*p < 0.05, significant difference vs. the control group.

### 3.4 Molecular docking studies

**Fig 6:**
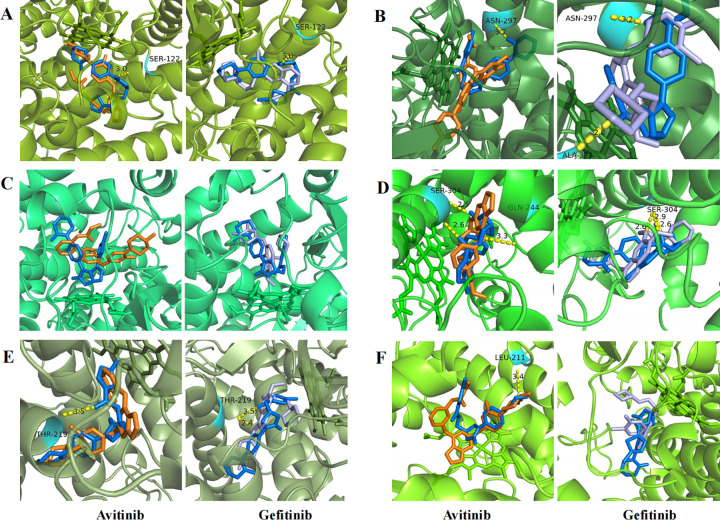
Molecular docking analysis of avitinib, gefitinib, and rivaroxaban on several CYP450 enzymes. Interaction sites of CYP1A2 (A), CYP2A6 (B), CYP2B6 (C), CYP2D6 (D), CYP2J2 (E) and CYP3A4 (F) enzyme with avitinib, gefitinib and rivaroxaban. The yellow dotted lines represent hydrogen bonding interactions between the small molecules and target proteins.

Molecular docking simulations (Fig 6) were carried out to analyze the mechanism of DDIs between avitinib, gefitinib and rivaroxaban in different CYP450 enzymes. The BE of small molecules with different CYP450 enzymes was presented in Table 2. The BE less than -5.0 kcal/mol indicates a strong binding interaction between the small molecule and target protein [29]. CYP3A4 and CYP2D6 have stronger binding ability with rivaroxaban, with BE of -8.1 kcal/mol and -8.8 kcal/mol, respectively. Avitinib and rivaroxaban bind closely with CYP3A4 in the same binding pocket (Fig 6D), with BE of -8.8 kcal/mol. Gefitinib and rivaroxaban bind closely with several enzymes and CYP2D6 showed the strongest BE of -10.1 kcal/mol (Fig 6B). According to the molecular docking results, avitinib and gefitinib mainly inhibit the binding of rivaroxaban with CYP3A4 and CYP2D6 by spatial hindrance, thereby inhibiting the metabolism of rivaroxaban.

**Table 2:** The binding energy of small molecules with different CYP450 enzymes.

CYP450 enzyme	Rivaroxaban (kcal/mol)	Avitinib (kcal/mol)	Gefitinib (kcal/mol)
CYP1A2	-5.7	-0.2	-7.2
CYP2A6	13.4	26.1	8.2
CYP2B6	-0.9	2.9	-7.3
CYP2D6	-8.8	-4.6	-10.1
CYP2J2	-4.7	-2.2	-6.1
CYP3A4	-8.1	-8.8	-7.9

## 4 Discussion

Cancer has been shown to induce a hypercoagulable state, thereby increasing the risk of VTE, which in turn exacerbates the disease burden in patients with cancer and thus increases morbidity and mortality [1,10]. International guidelines, supported by mounting clinical evidence, have endorsed the use of DOACs as anticoagulants for VTE in patients with cancer [1,2,10]. Rivaroxaban, one of the most widely used DOACs in clinical practice, exerts its effects by directly inhibiting coagulation factor Xa [8,30]. The transport and metabolism of rivaroxaban are influenced by various enzymes, including P-gp, BCRP, CYP2J2, CYP3A4 and CYP2D6 [13–16,31]. Notably, these enzymes are targets of potential DDIs. Drug combinations could result in enhancement or inhibition of enzyme activity, which may lead to significant differences in the plasma concentration of rivaroxaban [31,32]. In a study of patients receiving anticoagulant therapy with rivaroxaban, combination with clarithromycin, a P-gp and CYP3A4 inhibitor, led to a two-fold increase in the AUC value for rivaroxaban [33]. In 2022, Zhang et al. showed that polymorphisms in the ABCB1 gene encoding P-gp were highly associated with dose-adjusted rivaroxaban peak concentration in plasma [34]. The changes in rivaroxaban blood concentrations may affect the drug’s effects and lead to embolism or hemorrhage events. A multicenter prospective cohort study in 2023 showed that rivaroxaban peak concentration was associated with risk of bleeding events in patients with atrial fibrillation [35].

TKIs have been extensively used to treat neoplastic diseases [18,19]. However, patients with cancer who receive TKIs may develop DDIs when undergoing rivaroxaban anticoagulant therapy, owing to the involvement of common enzymes in the transport and metabolic pathways [21,22]. In recent years, there has been growing evidence highlighting the extensive DDIs between TKIs and DOACs [21,23,24]. Numerous *in vivo* and *in vitro* studies have substantiated the impact of various TKIs on the metabolism of rivaroxaban [21,23–25]. CYP450 enzymes and transporters jointly play important roles in the pharmacokinetic association [25]. Wang et al. reported in 2022 that two types of TKIs, avitinib and osimertinib, exhibited a concentration-dependent inhibition of rivaroxaban hydroxylation by CYP2J2 *in vitro* [24]. In 2022, Lafaie et al. assessed the potential DDIs mediated by P-gp between 11 types of TKIs with DOACs *in vitro*. The inhibitory properties of the various TKIs on DOACs were assessed using the IC_50_ value. Eight of the TKIs had IC_50_ values below 100 μM and IC_50_ values of less than 10 μM were observed with the combination of erlotinib, nilotinib with rivaroxaban. The bleeding risk is greater with rivaroxaban with lower IC_50_ values [21]. The potential impact of various TKIs on the metabolism of rivaroxaban may result in either inhibition or promotion. In a study conducted by Zhao et al. in 2022 [25], imatinib inhibited rivaroxaban metabolism and significantly increased rivaroxaban C_max_ and AUC (by 90.43% and 119.96%, respectively). Conversely, the combination of sunitinib and rivaroxaban resulted in a 62.32% reduction in exposure to rivaroxaban *in vivo*. These findings suggested a widespread risk of DDIs between TKIs and rivaroxaban. However, there is a dearth of comprehensive assessment regarding the DDIs between rivaroxaban and multiple EGFR TKIs, which are extensively prescribed to patients with NSCLC [20].

Here, the effects of various EGFR TKIs on rivaroxaban metabolism were evaluated, and avitinib and gefitinib showed the most potent inhibitory effects. In *in vitro* studies on RLMs, we observed that most EGFR TKIs inhibited rivaroxaban metabolite production to varying degrees, indicating that the co-administration of these drugs may increase the risk of anticoagulant bleeding in individuals taking rivaroxaban. Moreover, there may be differences in the inhibition patterns of rivaroxaban by different TKIs. Wang et al. found that osimertinib demonstrated competitive inhibition when rivaroxaban was used as the probe substrate, while a two-site kinetic model was established to explain the atypical inhibitory kinetics of avitinib [24]. In this study, avitinib and gefitinib strongly inhibited rivaroxaban activity, and were confirmed as mixed inhibitors of rivaroxaban; both exhibited IC_50_ < 10 μM, implying a higher risk of DDIs. Gefitinib, the first TKI approved for the treatment of NSCLC, has been shown to significantly prolong progression-free survival [36]. Zhao et al. [25] found that gefitinib potently inhibited CYP2J2- and CYP3A4-mediated rivaroxaban metabolism *in vitro*. However, subsequent *in vivo* investigations failed to observe any effects of gefitinib on the pharmacokinetic parameters of rivaroxaban [25]. Our study demonstrated significant inhibition of rivaroxaban metabolism by gefitinib *in vitro* and *in vivo*. Gefitinib significantly increased rivaroxaban’s AUC, C_max_, and T_max_, but it significantly reduced its V_z_/F and CL_z_/F. In the gefitinib group, the significant increase in the MRT indicated an extended drug activity *in vivo*. Avitinib is a third-generation EGFR-TKI small-molecule inhibitor [37]. To date, no studies have been reported on DDIs between avitinib and rivaroxaban. The *in vitro* experiments conducted by Shi et al. [38] revealed different inhibitory effects of avitinib on six probe substrates. Subsequent *in vivo* studies indicated that this inhibition could be attributed to the effect of avitinib on CYP450 enzymes [38]. Therefore, attention should be paid to DDIs when avitinib is co-administered with other medications metabolized by CYP450 enzymes. Our study revealed significant inhibition of rivaroxaban’s pharmacokinetics by avitinib and gefitinib. Consequently, it is imperative to give considerable attention to the potential bleeding risk resulting from DDIs in patients co-administered with rivaroxaban and EGFR TKIs, particularly avitinib and gefitinib.

CYP450 enzymes play a role in the hydroxylation of rivaroxaban and the metabolism of EGFR TKIs. To further investigate the mechanisms leading to DDIs between avitinib, gefitinib and rivaroxaban, several key enzymes involved in the hydroxylation of rivaroxaban were selected for molecular docking verification [27]. Molecular docking simulations offer valuable insights into the structural and energetic characteristics of the compound-target associations, aiding in the understanding of molecular structure-based mechanisms of DDIs [39–41]. The BE less than -5.0 kcal/mol indicates a strong binding affinity. Molecular docking analysis showed that many CYP450 enzymes affected the metabolic process of rivaroxaban. Avitinib predominantly impacts rivaroxaban metabolism via CYP3A4, forming hydrogen bonds with catalytic residues LEU 211. This interaction likely obstructs rivaroxaban’s access to the CYP3A4 active site, explaining the mixed inhibition observed in Lineweaver-Burk plots. Gefitinib can affect rivaroxaban metabolism through multiple CYP450 enzymes, particularly by obstructing the binding site of rivaroxaban and CYP2D6. The combined effects of multiple enzymes and stronger binding affinity may account for the more pronounced inhibitory effect and significant alterations in pharmacokinetic parameters observed in the gefitinib group during both in vitro and in vivo experiments.

Our study has some limitations. First, we focused on demonstrating the inhibitory effects of avitinib and gefitinib on the metabolism of rivaroxaban. However, further investigation is required to determine the impact of rivaroxaban on the metabolism of EGFR TKIs, to fully understand the potential DDIs. Second, our study only detected the presence of M-2, the major metabolite of rivaroxaban. It is important to note that previous studies have identified other metabolites, such as M-1 and M-3, in the metabolism of rivaroxaban [42]. Therefore, simultaneous detection of these metabolites would provide valuable insights into the influence of avitinib and gefitinib on rivaroxaban metabolism. Third, we only investigated the role of the CYP450 enzyme in the DDIs through molecular docking studies. Further enzyme functional studies *in vitro* and *in vivo* are needed to evaluate the impact of CYP450 enzymes and drug transporters. Finally, all experiments were performed in rats, and further studies in humans are required to gain a more comprehensive understanding of the clinical interaction between EGFR TKIs and rivaroxaban.

## 5 Conclusion

Overall, TKIs and rivaroxaban are often co-administered in cancer patients with VTE. Our results indicate that several EGFR TKIs, particularly avitinib and gefitinib, exhibited DDIs in response to rivaroxaban. Administration of avitinib and gefitinib significantly increased rivaroxaban exposure, which may be due to the inhibition of drug-metabolizing enzymes. Consequently, patients with cancer treated with these TKIs may face an elevated risk of bleeding when undergoing anticoagulant therapy with rivaroxaban. The results of this study serve as a foundation for optimizing drug administration protocols for cancer patients with VTE. Further clinical trials are required to explore the potential DDIs between other TKIs and rivaroxaban and assess the impact of these DDIs on the prognosis and outcomes.

## Supporting information

S1 Data Raw data from *in vitro* experiments of avitinib and gefitinib groups, raw data from *in vitro* experiments of avitinib groups, raw data from *in vitro* experiments of gefitinib groups. (ZIP)
